# Food insecurity among Finnish private service sector workers: validity, prevalence and determinants

**DOI:** 10.1017/S1368980022000209

**Published:** 2022-04

**Authors:** Hanna M Walsh, Jaakko Nevalainen, Tiina Saari, Liisa Uusitalo, Turkka Näppilä, Ossi Rahkonen, Maijaliisa Erkkola

**Affiliations:** 1 Department of Food and Nutrition, University of Helsinki, Agnes Sjöbergin Katu 2, Helsinki 00014, Finland; 2 Health Sciences, Faculty of Social Sciences, Tampere University, Finland; 3 Work Research Centre, Faculty of Social Sciences, Tampere University, Finland; 4 Tampere University Library, Tampere University, Finland; 5 Department of Public Health, University of Helsinki, Finland

**Keywords:** Food security, Working poor, Occupational health, Income, Socio-economic factors

## Abstract

**Objective:**

To examine the prevalence and determinants of food insecurity among private sector service workers in Finland and assess validity of the Household Food Insecurity Access Scale (HFIAS) tool.

**Design:**

In this cross-sectional study, food insecurity and background characteristics were collected from Finnish private service workers via electronic questionnaires (2019) and national register data (2018–2019). We conducted univariate and multivariate logistic regression analyses to determine the variables explaining food insecurity. Validity of HFIAS was assessed with rotated principal component analysis and Cronbach’s *α*.

**Setting:**

Members of the trade union for private sector service workers, Service Union United (PAM), from all municipalities in Finland participated in the study in 2019.

**Participants:**

The subjects were 6435 private sector workers that were members of the Service Union United (PAM) in Finland. Mean age of participants was 44 years (sd 12·7 years).

**Results:**

Two-thirds of the participants (65 %) were food insecure with over a third (36 %) reporting severe food insecurity. Reporting great difficulties in covering household expenses and young age markedly increased the risk of severe food insecurity (OR 15·05; 95 % CI 10·60, 21·38 and OR 5·07; 95 % CI 3·94, 6·52, respectively). Not being married, low education, working in the hospitality industry, being male and living in rented housing also increased the probability of severe food insecurity. The HFIAS tool demonstrated acceptable construct and criterion validity.

**Conclusions:**

Severe food insecurity was widespread and associated with low socio-economic status, young age and being male among Finnish private sector service workers, emphasising the need for regular monitoring of food insecurity in Finland.

Limited systematic data on food insecurity levels in Europe exist, whereas the USA and Canada are the few high-income countries that have regularly monitored national food insecurity levels within the population since the 1990s^(1)^. Yet, there are reports of rising numbers of people looking for emergency food support in Europe^(2)^. The sporadic studies measuring food insecurity in Europe have found it exists^(3–6)^, even in countries often characterised as having social-democratic welfare regimes such as the Nordic countries^(7,8)^.

There is some research on the entrenchment of food aid and charity in Finland^(9,10)^, yet there are little data on national levels of food insecurity. In a 2017 FAO report^(11,12)^, the prevalence of moderate and severe food insecurity in Finland (8·3 %) was higher than that of any other Nordic country (Sweden, Norway, Denmark, Iceland: moderate and severe food insecurity 5·1–6·7 %) and that of the United Kingdom (5·6 %). According to a 2001 Finnish study examining food insecurity, 11 % of a nationally representative sample reported experiences of running out of money to buy food, 9 % reported fears of running out of food due to economic problems and 3 % had had too little food due to lack of money^(13)^.

In Finland, low household income, recent unemployment and economic problems in childhood were all associated with food insecurity^(13)^. There are similar findings worldwide, as food insecurity has been found to be a consequence of multiple economic and resource issues such as lower household income^(5,7,8,14–16)^, lack of assets and savings^(17)^ and income instability^(18,19)^. Other vulnerabilities such as receiving disability pensions/benefits^(7,20)^, being a single-parent household^(6,7)^, having lower education^(3,5)^, being an immigrant or asylum seeker^(8,21,22)^ and renting housing^(14)^ have also been associated with food insecurity. Food insecurity has also been linked with unhealthy diets^(4,6,7,23)^ and lower nutrients intake^(24)^. A range of health outcomes have also been linked to food insecurity, including higher mortality^(25,26)^, higher prevalence of chronic conditions such as hypertension^(27)^, diabetes^(28)^, arthritis and back problems^(29)^, mental health problems including depression and stress^(30)^ and mood and anxiety disorders^(29)^. Thus unsurprisingly, household food insecurity has also been found to predict increased universal health care utilisation and costs of working-age adults^(31)^.

A vulnerable group in Finland are the workers in the private service sector where many of the characteristics associated with higher risk of in-work poverty accumulate such as part-time employment, immigrant background, low education and being female^(32,33)^. Due to the high prevalence of trade union membership in Finland, recruiting participants via trade unions can offer a new way of reaching a hard-to-survey population group. Finland had the fourth highest trade union density of OECD member countries in 2018^(34)^ and according to the Ministry of Economic Affairs and Employment of Finland, the trade union membership rate among private service sector workers was 48 % in Finland in 2017^(35)^.

Thus, the aim of this study was to inspect the prevalence of food insecurity among private service sector union members in Finland during 2019 and identify the main socio-demographic, economic, health and work-related factors associated with food insecurity. To our knowledge, no food insecurity measure has been validated in Finland before and thus we will also inspect the validity of the food insecurity measurement tool among the private service sector union members in Finland.

## Subjects and methods

### Study design

The data were collected in collaboration with the Finnish Service Union United (PAM). PAM has almost 210 000 members, 76 % of them women, working in retail trade, property services, security services as well as tourism, restaurant and leisure services^(36)^.

Data were collected during April and May 2019 via an online Study Survey form. The invitation to the study was sent to 111 850 PAM members, that is, to all Finnish-speaking employed, unemployed and retired members who had provided their email address in the PAM member register (student members were excluded) (online supplementary material, Supplement 1). The Study Survey included questions on food insecurity and background characteristics. After this, PAM’s own annual Member Survey, including work-related questions, was similarly sent (May–June 2019) to PAM members (110 833) via email.

Participants were asked for permission to link their survey answers with national register data provided by Statistics Finland for the years 2018–2019. Data obtained from Statistics Finland from 2019 included sex, year of birth, municipality type, region of residence, as well as income and income transfers from 2018.

### Participants

The Study Survey was initially answered by 6573 participants (6·5 % of those invited) while the Member Survey was answered by 6528 (6·5 %) participants. Once those who had denied use of their data for study purposes were deleted, erroneous ID were fixed and participants that did not have a national identification number in the background data were deleted, data were available for 6435 for the Study Survey and 6375 for the Member Survey of which 3998 participants had answered both surveys. National register data from the years 2018 and 2019 were available for 6431 and 6421 participants, respectively, of the 6435 who had answered the Study Survey.

### Food insecurity

Food insecurity has previously been measured in Finland using the Food Insecurity Experience Scale^(11)^, a collection of questions based on the Edmonton Food Policy Council’s survey^(13)^, and a modified version of the Household Food Insecurity Access Scale (HFIAS) among food aid recipients^(37)^, all of which measured food insecurity on an individual level. Due to availability of the translated version used and to enable comparison between studies, we selected the modified HFIAS questionnaire for our study.

The HFIAS tool has been developed by the USAID’s Food and Nutrition Technical Assistance project^(38)^ based on a thorough review of the commonalities found across different cultures in qualitative food insecurity studies, thus allowing it to distinguish food insecure from food secure households across different cultural contexts. The HFIAS questionnaire (online supplementary material, Supplement 2) was first translated to Finnish and adapted in cooperation with the Finnish Blue-Ribbon association for a recent study of 129 participants receiving food aid^(37)^. As discussed by Coates *et al*.^(38)^, the concept of ‘household’ is highly context specific and should be defined uniformly for participants. Due to the nature of the participants, many of whom were homeless, the questionnaire was modified to focus on the individual experience of food insecurity due to the fact that ‘household’ may not have been a relevant unit for the participants. The modified questionnaire was then piloted among nine residents of a supported housing service unit for homeless adults. All nine were able to answer the questionnaire well and no modifications were made.

We felt that individual-level measurements were relevant for this study not only to enable comparison with previous research in Finland^(11,13,37)^, but also because the HFIAS was administered as an online survey, meaning we could not ensure that the respondent was the person in the household who was most involved with food preparation and meals, as advised by Coates *et al.* ^(38)^. Furthermore, the focus on household-level food insecurity has been questioned as it assumes a standard model where there is one decision-maker who always acts for the benefit of the household, where resources are pooled and where worry about food is a collective experience^(39)^. Research has shown that, in reality, power imbalances and differences in domains of responsibility exist within households across the world and resources are not always distributed equitably, nor are experiences of food insecurity the same^(39,40)^. The experience may differ especially between men and women due to the gendered roles in food acquisition, preparation and providing income to buy food. For example, Coates *et al.* ^(39)^ found that nearly one-third of Bangladeshi households were classified into different food security categories using female *v*. male responses to the questions. Furthermore, O’Connell and Brannen^(41)^ stated that it is often mothers who go without food to prioritise the needs of children and male partners. Thus, Coates *et al.* ^(39)^ and FAO^(11)^ suggest using individual-level food insecurity analyses which will allow inspection of intra-household differences too.

PAM members answered the nine HFIAS questions as part of the online Study Survey, based on which, participants were then categorised as food secure or mildly, moderately or severely food insecure, as instructed by Coates *et al.* ^(38)^. The HFIAS questions ask if participants have experienced issues related to worry about having enough food, limited food quality and limited food quantity and how often (rarely, sometimes, often) they have experienced these issues in the last 30 d. Thus, classification into the different food insecurity categories depends on affirmative answers to certain questions as well as frequency of the experience, as explained by Coates *et al.* ^(38)^.

### Background and work-related characteristics

Variables obtained from the Study Survey included self-reported height and weight (used to calculate BMI), highest obtained education level, marital status, household size, number of children under 18 in the household, type of housing, employment status, self-assessed adequacy of income compared with expenses and self-perceived health status. The industry of employment was obtained from the Member Survey. All data were self-reported in the Study and Member Surveys. Variables obtained from national register data provided by Statistics Finland included sex (2019), year of birth from which age was calculated (2019), type of municipality (2019), region of residence (2019), individual earned income in state taxation^(42)^ during the year 2018 and received income transfers^(43)^ during the year 2018.

### Analysis

The accuracy of the modified HFIAS tool was evaluated by examining content, construct and criterion validity. *Content validity* refers to the extent to which the items on a test are representative of the entire domain the test seeks to measure. Thus, we evaluated whether the nine questions of HFIAS covered all aspects of food security in a Finnish context, based on previous literature. *Construct validity* is the degree to which a tool measures what it is supposed to measure. Thus, we evaluated whether HFIAS has a multidimensional construct and what dimensions of food insecurity it measures in the Finnish context. This was evaluated by factor analysis (rotated principal component analysis) as done in previous studies^(44–46)^. The factors were computed from the correlation matrix and oblique rotation (Oblimin with Kaiser normalisation) was selected because the different domains of food insecurity may be correlated. The number of factors was determined by the scree plot and those with an eigenvalue > 1. The internal consistency of the scale and domains revealed in the factor analysis of the tool were measured by Cronbach’s *α*.

*Criterion validity* measures how well one measure predicts an outcome of another measure. The criterion validity of the HFIAS tool was investigated by looking at whether it distinguished between different socio-demographic groups with established differences in food insecurity such as sex, age, family structure, housing, income, education, living area, marital status and occupation^(7,14,44)^. The associations between the food insecurity levels and socio-demographic variables were assessed with chi-squared tests.

Univariate binary logistic regression was used to explore which individual variables increased the risk of severe food insecurity. Food insecurity was made into a dichotomous variable where the food secure, mildly and moderately food insecure categories were combined and the severely food insecure category was compared with this group. Self-perceived health status and BMI were not investigated due to the uncertainty in causal direction. We also conducted univariate multinomial logistic regressions to see how determinants were associated with the different levels of food insecurity.

Multiple explanatory variables were included into a multivariate binary logistic regression model explaining severe food insecurity to control for confounding and to obtain an adjusted estimate of the magnitude of the associations. The variables included in the model were based on which socio-demographic, economic and work-related variables were found to be associated with an increased risk of severe food insecurity in univariate logistic regression analyses. Income in state taxation was removed due to its collinearity with self-assessed adequacy of household income to cover expenses and the latter was deemed to be a better representation of the participants’ situation in 2019 and a stronger determinant. Received income transfers were removed from the model due to the challenges in interpreting its meaning as it includes a whole range of transfers including earnings-related and national pensions, other social security benefits (e.g. parental allowance) and social allowances (e.g. study grant)^(43)^. Household size correlated with marital status and was thus removed. Employment status was not significant in the model and was removed. The level of statistical significance used was 0·05. The participants with missing data on sex, age and education were excluded from the model. However, the participants with missing data on employment industry were included, due to there being so many (56 %). All statistical analyses were performed using the Statistical Package for the Social Sciences statistical software package version 27 (SPSS Inc.).

## Results

### Validity of the Household Food Insecurity Access Scale measure

The questions (1–3) related to milder forms of food insecurity received the most affirmative answers and the questions (7–9) relating to more severe levels of food insecurity received the most negative answers though the trend was not entirely consistent (e.g. questions one and six) (Table 1). The factor analysis of the nine HFIAS questions revealed two factors which explained 56·3 and 12·4 % of the total variance, respectively (Table 1). The first factor had high loadings on questions one to five reflecting a less severe form of food insecurity, while questions six to nine loaded onto the second factor, reflecting a more severe form of food insecurity (lacking food in quantity).

**Table 1:** Finnish Service Union United members’ response rates to each HFIAS question and each question’s factor loadings, 2019

		Response (%)				Factor loadings*	
	Modified HFIAS questions	No	Rarely	Sometimes	Often	First factor†	Second factor‡
1	Have you been worried about the adequacy of food?	67·3	16·1	12·4	4·3	**0·786**	0·087
2	Have you had to limit foods that you would have wanted to have eat?	55·6	18·9	16·8	8·7	**0·929**	−0·093
3	Have you had to eat more limitedly than you would have wanted?	51·6	16·8	19·0	12·5	**0·898**	−0·030
4	Have you had to eat foods that you did not want to eat?	68·6	19·0	9·5	2·9	**0·765**	0·029
5	Have you had to eat smaller portions than you would have wanted?	74·3	14·3	8·5	2·9	**0·632**	0·262
6	Have you had to skip meals?	70·1	15·0	10·6	4·4	0·248	**0·640**
7	Have you been in a situation where you had no food to eat?	81·1	12·2	5·1	1·6	0·200	**0·695**
8	Have you gone to sleep hungry?	73·5	17·3	7·1	2·1	0·133	**0·747**
9	Have you gone the whole day without eating?	84·3	10·2	4·3	1·1	−0·187	**0·909**

HFIAS, Household Food Insecurity Access Scale.

* Factor analysis (rotated principal component analysis), Kaiser–Meyer–Olkin measure of sampling adequacy = 0·912, factor loadings greater than 0·6 are indicated in bold font.

† Eigenvalue = 5·071.

‡ Eigenvalue = 1·119.

Cronbach’s *α* of the scale was 0·899 which indicated good internal consistency. The subscales had satisfactory internal consistency too as Cronbach’s *α* for the first factor was 0·888 and 0·816 for the second factor.

### Participants’ characteristics

The food security levels were calculated for 6435 participants, but socio-demographic and work-related details were not available for all participants. The majority of the participants were women (80 %), and the mean age of all participants was 44 years (sd 12·7 years) ranging from 17 to 83 years (Table 2). Most participants had completed upper secondary school, vocational education, obligatory education or less as their highest level of education (83 %). Being married or cohabiting were the most common situations and correspondingly most people lived with at least one other person. The majority of participants did not have any children under 18 years in their households (68 %). Over half of the participants lived in housing they owned (57 %) and the majority lived in urban areas (74 %). The region of residence was obtained for 6421 participants and they were spread out across all nineteen Finnish regions, the majority residing in Uusimaa (capital city region) (23 %), Pirkanmaa (Tampere region) (11 %), Southwest Finland (10 %) and Northern Ostrobothnia (8 %).

**Table 2:** The associations of socio-demographic and health-related variables with food insecurity among Finnish Service Union United members, 2019

	*n*	%	Food secure	Food insecure			*P* *
			%	Mild %	Moderate %	Severe %	
Overall prevalence	6435	100	35	12	17	36	
95 % CI			34·3, 36·6	10·8, 12·3	16·3, 18·2	34·6, 36·9	
Sex	6435	100					< 0·01
Female	5120	80	36	12	18	35	
Male	1301	20	35	10	15	40	
Missing data	14	0·2	29	7·1	14	50	
Age (years)	6435	100					< 0·01
17–29	987	15	22	11	14	52	
30–44	2214	34	31	11	18	39	
45–59	2381	37	39	12	19	31	
60+	839	13	54	12	15	20	
Missing data	14	0·2	29	7·1	14	50	
Highest education	6435	100					0·01
Obligatory education or less	708	11	36	11	14	39	
Upper secondary school or vocational	4655	72	35	11	18	36	
Undergraduate	964	15	38	13	18	31	
Postgraduate	104	1·6	41	13	21	25	
Missing data	4	0·1	50	0	0	50	
Marital status	6435	100					< 0·01
Married or registered partnership	2523	39	43	12	18	27	
Cohabitation	1785	28	35	12	16	37	
Divorced or separated	597	9·3	28	11	20	41	
Widow	97	1·5	40	11	15	33	
Single	1433	22	26	9·9	17	48	
Household size	6435	100					< 0·01
1	1769	27	30	11	17	42	
2	2571	40	41	11	15	32	
3	915	14	32	11	19	38	
4	827	13	34	14	21	31	
5+	353	5·5	30	13	25	32	
Number of children under 18 years in the HH	6435	100					< 0·01
0	4394	68	37	11	16	35	
1	934	15	31	12	19	39	
2	743	12	33	13	19	35	
3	247	3·8	30	14	26	31	
4+	88	1·4	23	11	25	41	
Missing data	29	0·5	38	10	10	41	
Housing	6435	100					< 0·01
Owner-occupied housing	3661	57	42	12	17	29	
Right of occupancy housing	183	2·8	37	8·7	19	36	
Rented municipal housing	719	11	28	10	20	42	
Other rented dwelling or company housing	1863	29	26	11	17	47	
Supported housing	2	0·03	0	0	0	100	
Homeless	7	0·1	0	14	14	71	
Municipality type	6435	100					0·42
Urban	4755	74	35	11	17	36	
Semi-rural	929	14	35	12	19	33	
Rural	737	11	38	12	15	35	
Missing data	14	0·2	29	7·1	14	50	
BMI	6435	100					< 0·01
< 18·5	79	1·2	34	8	8	51	
18·5–24·99	2263	35	36	12	16	36	
25–29·99	2124	33	38	12	17	34	
≥ 30	1927	30	32	11	17	37	
Missing data	42	0·1	38	12	19	31	
Self-perceived health status	6435	100					< 0·01
Good	1892	29	42	12	15	31	
Quite good	2517	39	35	12	17	35	
Average	1540	24	31	11	19	40	
Quite poor	414	6·4	26	8·2	22	43	
Poor	72	1·1	28	11	14	47	

HH, household.

* Chi-squared test

The majority of the participants were employed (70 %) and the mean earned income in state taxation was 2043 €/month (sd 746 €/month) ranging from 0 to 7450 €/month (Table 3). Employment industry was obtained for 44 % of the participants. Of those who replied, the majority worked in the retail industry (50 %), followed by those in hospitality (20 %) and then those in property maintenance (12 %). One-third of the participants received minimal (0–9 €/month) income transfers from the government, while the rest received 10–5701 €/month, the overall median being 200 €/month.

**Table 3:** The associations of economic and work-related variables with food insecurity among Finnish Service Union United members, 2018–2019

	*n*	%	Food secure	Food insecure			*P* *
				Mild	Moderate	Severe	
			%	%	%	%	
Overall prevalence	6435	100	35	12	17	36	
95 % CI			34·3, 36·6	10·8, 12·3	16·3, 18·2	34·6, 36·9	
Employment status	6435	100					< 0·01
Employed	4529	70	37	11	17	34	
Partly working, partly retired	136	2·1	40	9·6	19	31	
Laid-off	12	0·2	25	17	8·3	50	
Unemployed	579	9·0	30	9·5	16	45	
Parental leave/stay at home parent	174	2·7	33	14	18	36	
Long-term sick leave (over 6 months)	107	1·7	33	3·7	21	42	
Retired	301	4·7	50	12	14	24	
Not working for other reasons	29	0·5	17	21	24	38	
Other	568	8·8	24	15	18	43	
Employment industry	6435	100					< 0·01
Retail	1393	22	39	2·5	19	30	
Hospitality	570	8·9	39	8·8	16	37	
Property maintenance	345	5·4	36	9·3	20	35	
Security	121	1·9	38	9·9	20	32	
Hairdressing	28	0·4	36	14	14	36	
Other	347	5·4	40	12	17	31	
Missing data	3631	56	33	12	17	39	
Earned income in state taxation (monthly)†	6435	100					< 0·01
0–999 €	509	9·4	23	12	17	49	
1000–1599 €	1249	19	30	11	17	42	
1600–1999 €	1210	19	33	12	19	36	
2000–2499 €	1891	29	36	13	18	33	
2500 €+	1570	24	44	11	16	29	
Missing data	6	0·1	50	0	17	33	
Current transfers received (monthly)†	6435	100					< 0·01
0–9 €	2091	32	42	12	15	31	
10–199 €	1259	20	35	13	19	33	
200–399 €	661	10	33	12	17	39	
400–799 €	967	15	29	12	18	41	
800–1200 €	658	10	32	9	20	38	
>1200 €	793	12	32	10	17	41	
Missing data	6	0·1	50	0	17	33	
How well can households cover expenses with income?	6435	100					< 0·01
With great difficulty	387	6·0	7·2	3·9	16	73	
With difficulty	784	12	9·3	7·0	24	59	
With small difficulties	1837	29	22	14	23	41	
Quite easily	1820	28	44	13	15	27	
Easily	1130	18	51	12	12	21	
Very easily	477	7	70	8·6	6·1	15	

* Chi-squared test.

† Data from 2018, all other data from 2019.

### Prevalence and determinants of food insecurity

Of the 6435 respondents, over a third (36 %) were severely food insecure, 29 % were mildly or moderately food insecure and a third (35 %) were food secure (Table 2). Tables 2 and 3 present the distribution of food insecurity status by socio-demographic, health, economic and work-related variables. All variables apart from municipality type were associated with food insecurity levels.

### Explanatory variables of severe food insecurity in binary logistic analysis

In the univariate binary logistic regression analyses, being able to cover household expenses ‘with great difficulty’ and being young increased the probability of severe food insecurity markedly (online supplementary material, Supplements 3–4). Of the socio-demographic variables being single, divorced/separated or cohabiting, renting housing (or supported housing or being homeless), low educational level, a household size of one and three and being male increased the risk of being severely food insecure (online supplementary material, Supplement 3). Neither the number of children under 18 years in the household nor municipality type was associated with an increased risk of severe food insecurity. Of the economic and work-related variables, low income, high levels of income transfers from the government, being unemployed or laid off and working in hospitality and property maintenance also increased the probability of being severely food insecure (online supplementary material, Supplement 4).

### Explanatory variables of food insecurity levels in multinomial logistic analysis

In the univariate multinomial logistic regression analyses, the same determinants increased the risk of mild and moderate food insecurity as for severe food insecurity, apart from the following instances. Being male, having low education, being unemployed or laid off and working in hospitality did not increase risk of mild or moderate food insecurity (data not shown). In the binary analysis, having children under the age of 18 years was not associated with severe food insecurity. However, in the multinomial analysis, having children was associated with increased risks of mild, moderate and severe food insecurity: having any number of children, especially having three or more (compared with having none), increased the risk of mild and moderate food insecurity, whereas only having one child (compared with having none) increased the risk of severe food insecurity. Similarly, almost all family sizes (compared with a family size of two) increased the risk of mild and moderate food insecurity with large family sizes appearing to increase the risk the most in the multinomial model. A family size of five or more increased the risk of severe food insecurity in the multinomial model, as well as the family sizes of one and three also identified in the original binary logistic regression analysis. All levels of income transfers (compared with receiving 0–9 €/month) were associated with an increased risk of moderate and severe food insecurity, but only lower levels (10–199 and 400–700 €/month) were associated with an increased risk of mild food insecurity.

### Explanatory variables of severe food insecurity in multivariate binary logistic model

**Fig. 1:**
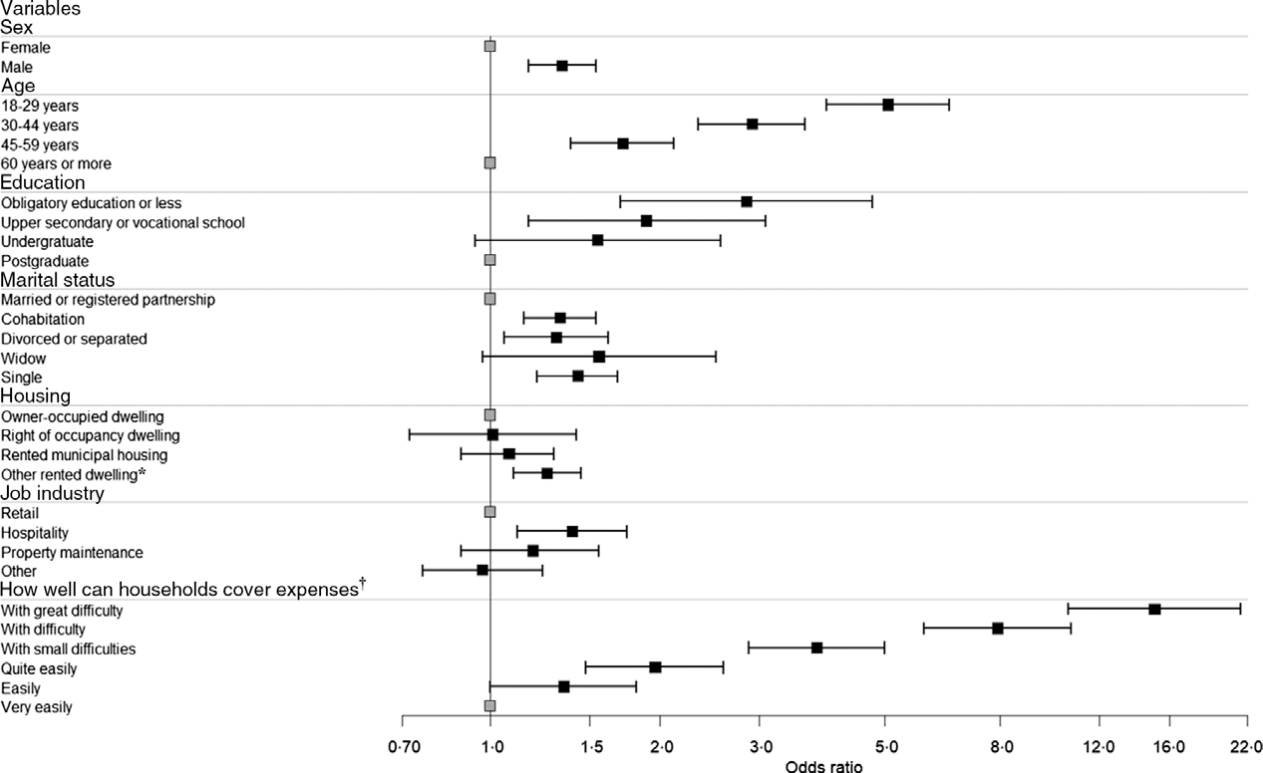
OR and CI for variables included in adjusted model explaining severe food insecurity among Finnish Service Union United members, 2019. *P* < 0·015 for all variables in the model, *n* 6417, Nagelkerke *R* ^2^ = 22 %. *Includes: other rented dwelling, company housing, supported housing and homeless. ^†^Full question: How well can household cover expenses in comparison with income

In the multivariate binary logistic regression model, being able to cover household expenses ‘with great difficulty’ and young age (18–29 years) retained their effect, markedly increasing the probability of severe food insecurity by 15- and 5-fold, respectively (Fig. 1). This is evident from the fact that 73 % of those ‘with great difficulty’ in covering expenses were severely food insecure, whereas 15 % of those who could ‘very easily’ cover expenses were severely food insecure (OR 15·05; 95 % CI 10·60, 21·85) (online supplementary material, Supplement 5). Half (52 %) of those in the youngest age bracket (18–29 years) were severely food insecure, while only a fifth (20 %) of those in the oldest age bracket (60+ years) were severely food insecure (OR 5·07; 95 % CI 3·94, 6·52). Having low education (OR 2·85; 95 % CI 1·70, 4·76), being single (OR 1·43; 95 % CI 1·21, 1·75), working in hospitality (OR 1·40; 95 % CI 1·12, 1·75), being male (OR 1·34; 95 % CI 1·17, 1·54) and living in rented housing (including company or supported housing or being homeless) (OR 1·24; 95 % CI 1·07, 1·43) also increased the risk of severe food insecurity.

## Discussion

The findings indicated that almost two-thirds of the private service sector workers were food insecure to some extent with over a third being severely food insecure. There are limited studies on the associations between individuals’ food insecurity levels and socio-demographic determinants, as most studies have looked at household-level food insecurity. However, the FAO worldwide study^(47)^ of 147 countries measured individual-level food insecurity using the Food Insecurity Experience Scale and compared country clusters. Similar to our results, they found that for the second cluster of ‘Rich and developed countries’ (including Finland), living without a partner (being single, widowed or divorced) and having a lower education level were associated with food insecurity. However, they found that sex was not significant in the second cluster, but in the first cluster of ‘Very rich and developed countries mainly outside Europe’, men were at more risk of food insecurity. Based on these comparisons, our results reflect decent criterion validity.

The HFIAS is designed to measure the three main domains found to constitute the household food insecurity experience: anxiety and uncertainty about the household food supply (question 1), insufficient quality (questions 2–4) and insufficient food intake and its physical consequences (questions 5–9). Arguably these domains are specific for household food insecurity^(48)^; however, Radimer *et al*.^(49)^ reported individual hunger to comprise four major components including intake insufficiency (a problem of intake quantity), diet inadequacy (a problem of intake quality) and disrupted eating patterns (not eating the three meals per day typical in high-income countries). The fourth, psychological, component was whether the person felt deprived and/or without eating choices. HFIAS covers the first two components mentioned. Furthermore, questions about having to skip meals or go a day without eating reflect the third component. The first question in HFIAS about worry reflects the fourth component to some extent. In addition, the Food Insecurity Experience Scale measure used on an individual level has been built on the research that informed HFIAS and contains very similar questions on worry and inadequate quality and quantity of food^(50,51)^.

To our knowledge, no factor analysis has been conducted on individual-level food insecurity measurement tools; hence, we can only make comparisons with studies that use the original household-level HFIAS. The factor analysis revealed two factors of milder and severer food insecurity somewhat reflecting two of the food insecurity domains (insufficient food quality and insufficient food quantity). The two factors explained 68·8 % of the variance similarly to previous studies^(44–46)^. As in previous studies^(44,45)^, we found that the anxiety and uncertainty domain did not appear as a separate construct of food insecurity, but rather as part of the ‘milder food insecurity’ factor. We found the fifth question (‘have you had to eat smaller portions than you would have wanted?’) also loaded onto the first factor (milder food insecurity), though it is part of the ‘insufficient food quantity’ domain. Our results were similar to those of the studies using household-level food insecurity measures^(44–46)^; however, more research is needed to understand the differences between the content and constructs of individual and household food insecurity.

The prevalence of food insecurity was higher in this study compared with the previous nationally representative studies conducted in Finland^(11,13)^, but lower than that found among Finnish food aid recipients^(37)^. This is in line with what can be expected, as many of the food aid recipients were unemployed and homeless, whereas, compared with the general population, our study sample had lower education and income levels^(52,53)^. Furthermore, Lund *et al*.^(7)^ found that interviews conducted via the internet more than doubled the prevalence of low/very low food security compared with computer-assisted telephone interviews. This may be due to people downplaying the severity of food insecurity to maintain social desirability. Face-to-face interviews and computer-assisted telephone interviews were used in the previous studies in Finland^(12,13)^, whereas in this study, the participants answered the survey online.

A weakness of this study is the low response rate, which raises questions about how representative the sample is. Based on statistics provided via email by PAM (A Veirto, Research Manager, personal communication, 29 November 2021), at the end of 2019, PAM had a slightly higher percentage of male members compared with the percentage in our study (online supplementary material, Supplement 6). Furthermore, the youngest age groups (under 31 years) and oldest (over 60 years) were under-represented in our study, which is partly not unexpected as student members were not surveyed. Of the 2804 (44 %) participants for whom the employment industry category was available, the percentages of participants working in retail, hospitality and security were very similar. Property maintenance was slightly under-represented, and the ‘Others’ group was over-represented. During 2019, 7 % of PAM members had listed their first language as other than Finnish and thus the survey was not sent to them. Hence, our study sample may not be completely representative of all PAM members, but it seems that Finnish-speaking non-student members were reasonably well captured. Because severe food insecurity was more common among men and young age groups in our study, and among immigrants in other studies^(21,22)^, it would seem more likely that the levels of food insecurity in this study were underestimates rather than overestimations of food insecurity levels in all PAM members. Furthermore, the fact that response rates are often lower among people of lower socio-economic status^(54)^ and food insecurity is higher among groups of lower socio-economic status further supports this interpretation. Moreover, trade union membership is lower among young people, men, the unemployed and those in part-time or fixed term contracts^(35)^ indicating that food insecurity may be even more prevalent among non-PAM members working in the private service sector.

Nevertheless, the multivariate model provides an adjusted estimate of the magnitude of the associations and can be used to indicate which groups within PAM members are at highest risk of being severely food insecure. Interestingly employment status was not significant in the model, possibly highlighting that work needs to be adequately compensated^(9,32,55)^. Alternatively, it could mean that the unemployed, laid-off and those on sick leave have adequate incomes to protect them from severe food insecurity considering most PAM union members would be on earning-related daily allowances rather than on basic social security. The latter has been found to be inadequate in covering reasonable minimum costs in Finland^(56)^. It must also be considered that the large majority of the participants were employed and that the unemployed, laid off and other groups may have been too small in size to be able to detect any statistically significant differences.

Respondents working in hospitality had a higher risk of severe food insecurity compared with retail workers. The number of young employees and employees with low educational level are especially emphasised in the restaurant and cleaning industries, respectively^(32)^. Jobs in the private service sector are characterised by part-time work, zero-hour and temporary contracts, and subcontracted work, especially in the hospitality industry^(32)^. Low work-intensity groups (those in part-time or temporary work) are experiencing increasingly higher poverty rates^(55)^ which are only expected to increase further due to the breakdown of collective agreements^(57,58)^ and the increasingly insufficient basic social security^(56,59)^ that the United Nations Committee on Economic, Social and Cultural Rights and the European Committee of Social Rights has warned Finland of^(60,61)^. Historically, in-work poverty has been comparatively low in Finland compared with other European countries due to the once relatively generous income transfer system resulting in the government essentially subsidising the inadequate incomes paid by the private sector^(32,55)^. Thus, one policy measure should be to ensure employers provide employees with adequate salaries and better contracts, especially paying attention to industries employing young and low education status groups, considering their increased risk of severe food insecurity. Furthermore, the adequacy of social security must be improved^(59)^. Considering the proportion of household expenditure spent on housing in all EU countries was highest in Finland^(62)^, providing more affordable housing, especially for those living alone, could also ensure improved livelihood as emphasised by the fact that rented housing was a risk factor for severe food insecurity. However, causality cannot be assumed from this study and renting may simply be associated with low wages and a lower socio-economic status.

Another alternative policy option to social security is universal basic income. A Finnish trial found that the basic income recipients’ perceptions of economic and health-related well-being and income were significantly better than that of the control group^(63,64)^. The recipients experienced significantly fewer problems related to health, stress and ability to concentrate^(63)^. They also had less issues with maintaining livelihood and reported themselves more happy^(64)^. This is relevant, as our results indicated that worse self-perceived health was associated with food insecurity.

Self-rated health has previously been found to be a strong predictor of mortality as well as associated with the number of physician contacts per year in Finnish populations^(65,66)^. Considering this and that food insecurity has been associated with multiple negative health outcomes in previous studies such as hypertension^(27)^, diabetes^(28)^, arthritis and back problems^(29)^, it is extremely worrying that over a third of the participants were severely food insecure and only a third were food secure. The association of BMI with food insecurity also highlights the seriousness of the situation people are facing. This could have major consequences for public health, for example, a Canadian study found that annual health care costs were 76 % higher in households with severe food insecurity compared with food-secure households^(31)^. Additionally, mental health problems have been found to be associated with food insecurity^(29,30)^ and they are also the main cause of premature retirement on a disability pension in Finland^(67)^.

There is a lack of reliable data and understanding of the extent of food insecurity and related public health implications in Finland to tackle the problem^(68)^. Our study focused on severe food insecurity, but it is important to understand the risk factors for mild and moderate food insecurity too, especially considering families with many children were at increased risk. Nevertheless, our findings show that food insecurity is a widespread issue among private sector service workers. The precariousness of employment, which can be seen in temporary contracts and jobs without a living wage, has increased in-work poverty^(9,32)^. This has resulted in the need for food charity, highlighting that food insecurity is a problem faced by many working people^(9,10)^. The Finnish Ministry of Social Affairs and Health is supporting charitable food aid financially, which is distributed by an unorganised and unregulated sector with unclear conditions^(9,69,70)^, to help citizens fulfil their basic needs. This is in direct contradiction to Finland identifying as a Nordic welfare state^(9,10)^. Finland along with other high-income countries should start monitoring food insecurity nationally on a regular basis, especially among vulnerable sections of the population.

## Supplementary material

For supplementary material accompanying this paper visit https://doi.org/10.1017/S1368980022000209. click here to view supplementary material
