# Supplementary material for: Food insecurity among Finnish private service sector workers: validity, prevalence and determinants
Source: Public Health Nutr. 2022 Jan 24;25(4):829–40. doi: 10.1017/S1368980022000209 (PMC9993037; doi:10.1017/S1368980022000209)
Supplement: Supplementary file 1 [file S1368980022000209sup001.zip › S1368980022000209sup001.pdf]

Supplement 3. Odds ratios and confidence intervals for sociodemographic and health-related variables explaining severe food insecurity among Finnish Service Union United members, 2019.

	N (%)	Severe food insecurity	
Covariate		OR*	95% CI
Sex	6421 (99.8)		
Female		1.00	
Male		1.27	1.12-1.44
Age (years)	6421 (99.8)		
17-29		4.41	3.57-5.44
30-44		2.60	2.15-3.14
45-59		1.78	1.47-2.15
60+		1.00	
Highest education	6431 (99.9)		
Obligatory education or less		1.92	1.20-3.06
Upper secondary school or vocational		1.72	1.10-2.69
Undergraduate		1.36	0.85-2.16
Postgraduate		1.00	
Marital status	6435 (100.0)		
Married or registered partnership		1.00	
Cohabitation		1.62	1.42-1.84
Divorced or separated		1.87	1.55-2.25
Widow		1.34	0.87-2.07
Single		2.48	2.17-2.84
Household size	6435 (100.0)		
1		1.53	1.35-1.74
2		1.00	
3		1.30	1.11-1.52
4		0.95	0.80-1.13
5+		1.00	0.79-1.27
Number of children under 18 years in the HH	6406 (99.5)		
0		1.00	
1		1.18	1.02-1.36
2		0.99	0.84-1.16
3+		0.92	0.73-1.17
Housing	6435 (100.0)		
Owner-occupied dwelling		1.00	
Right of occupancy dwelling		1.37	1.00-1.86
Rented municipal housing		1.78	1.51-2.09
Other rented dwelling, company housing, supported housing or homeless		2.21	1.97-2.48
Municipality type	6421 (99.8)		
Urban		1.072	0.911-1.261
Semi-rural		0.936	0.764-1.148
Rural		1.00	

HH, household

*Univariate binary logistic regression analysis
