# Supplementary material for: Food insecurity among Finnish private service sector workers: validity, prevalence and determinants
Source: Public Health Nutr. 2022 Jan 24;25(4):829–40. doi: 10.1017/S1368980022000209 (PMC9993037; doi:10.1017/S1368980022000209)
Supplement: Supplementary file 1 [file S1368980022000209sup001.zip › S1368980022000209sup002.pdf]

Supplement 2. Modified and translated Finnish Household Food Insecurity Access Scale (HFIAS) questions compared to original HFIAS questions, 2019.

	**Original HFIAS questions**	**HFIAS questions translated to Finnish and adjusted to address only the respondent not the whole household.**	**Finnish HFIAS questions translated back to English**
Mode	Face-to-face interview	Online questionnaire	
Intro	In the past four weeks…	Ajattele edellistä kuukautta. Oletko kohdannut seuraavia ruoan riittävyyteen liittyviä ongelmia, jotka johtuvat taloudellisista syistä:	Think about the previous month. Have you faced any of the following problems related to food sufficiency due to financial reasons:
1	Did you worry that your household would not have enough food?	Oletko ollut huolissasi ruoan riittävyydestä?	Have you been worried about the sufficiency of food?
2	Were you or any household member not able to eat the kinds of foods you preferred because of a lack of resources?	Oletko joutunut olemaan syömättä ruokia, joita olisit halunnut syödä?	Have you had to limit foods that you would have wanted to have eat?
3	Did you or any household member have to eat a limited variety of foods due to a lack of resources?	Oletko joutunut syömään yksipuolisemmin kuin olisit halunnut?	Have you had to eat more limitedly than you would have wanted?
4	Did you or any household member have to eat some foods that you really did not want to eat because of a lack of resources to obtain other types of food?	Oletko joutunut syömään ruokia, joita et olisi halunnut syödä?	Have you had to eat foods that you did not want to eat?
5	Did you or any household member have to eat a smaller meal than you felt you needed because there was not enough food?	Oletko joutunut syömään pienempiä annoksia kuin olisit halunnut?	Have you had to eat smaller portions than you would have wanted?
6	Did you or any household member have to eat fewer meals in a day because there was not enough food?	Oletko joutunut jättämään aterioita väliin?	Have you had to skip meals?
7	Was there ever no food to eat of any kind in your household because of lack of resources to get food?	Onko sinulla ollut tilannetta, että sinulla ei ole ollut mitään syötävää?	Have you been in a situation where you had no food to eat?
8	Did you or any household member go to sleep at night hungry because there was not enough food?	Oletko mennyt nälkäisenä nukkumaan?	Have you gone to sleep hungry?
9	Did you or any household member go a whole day and night without eating anything because there was not enough food?	Oletko ollut koko päivän syömättä?	Have you gone the whole day without eating?
Reply	No (*Skip the follow up question*)/Yes	En<br>Harvoin (1-2 kertaa/kk)<br>Joskus (3-10 kertaa/kk)<br>Usein (yli 10 kertaa/kk)	No<br>Rarely (1-2 times/month)<br>Sometimes (3-10 times/month)<br>Often (over 10 times/month)
Follow up question	How often did this happen?<br>1 = Rarely (once or twice in the past four weeks)<br>2 = Sometimes (three to ten times in the past four weeks)<br>3 = Often (more than ten times in the past four weeks)	-	-
