# Supplementary material for: Food insecurity among Finnish private service sector workers: validity, prevalence and determinants
Source: Public Health Nutr. 2022 Jan 24;25(4):829–40. doi: 10.1017/S1368980022000209 (PMC9993037; doi:10.1017/S1368980022000209)
Supplement: Supplementary file 1 [file S1368980022000209sup001.zip › S1368980022000209sup003.pdf]

Supplement 5. Odds ratios and confidence intervals for variables included in adjusted model* explaining severe food insecurity among Finnish Service Union United members, 2019. $p < 0.015$ for all variables in the model, N = 6,417, Nagelkerke R^2 = 22%.

Covariate	N	Severe food insecurity	
		OR	95% CI
	6417 (99.7%)		
Sex			
Female		1.00	
Male		1.34	1.17-1.54
Age (years)			
17-29		5.07	3.94-6.52
30-44		2.91	2.34-3.61
45-59		1.72	1.39-2.12
60+		1.00	
Highest education			
Obligatory education or less		2.85	1.70-4.76
Upper secondary school or vocational		1.89	1.17-3.08
Undergraduate		1.55	0.94-2.56
Postgraduate		1.00	
Marital status			
Married or registered partnership		1.00	
Cohabitation		1.33	1.15-1.54
Divorced or separated		1.31	1.06-1.62
Widow		1.56	0.97-2.51
Single		1.43	1.21-1.68
Housing			
Owner-occupied dwelling		1.00	
Right of occupancy dwelling		1.01	0.72-1.42
Rented municipal housing		1.08	0.89-1.30
Other rented housing, company housing, supported housing or homeless		1.26	1.10-1.45
Employment industry			
Retail		1.00	
Hospitality		1.40	1.12-1.75
Property maintenance		1.19	0.90-1.56
Other (including security and hairdressing)		0.97	0.76-1.24
Missing data		1.24	1.07-1.43
How well can households cover expenses with income?			
With great difficulty		15.05	10.60-21.38
With difficulty		7.93	5.87-10.71
With small difficulties		3.79	2.87-5.00
Quite easily		1.96	1.48-2.59
Easily		1.35	1.00-1.82
Very easily		1.00	

*Multivariate binary logistic regression model
