# Supplementary material for: Food insecurity among Finnish private service sector workers: validity, prevalence and determinants
Source: Public Health Nutr. 2022 Jan 24;25(4):829–40. doi: 10.1017/S1368980022000209 (PMC9993037; doi:10.1017/S1368980022000209)
Supplement: Supplementary file 1 [file S1368980022000209sup001.zip › S1368980022000209sup005.pdf]

Supplement 4. Odds ratios and confidence intervals for economic and work-related variables explaining severe food insecurity among Finnish Service Union United members, 2018-2019.

	N (%)	Severe food insecurity	
Covariate		OR*	95% CI
Employment status	6435 (100)		
Employed		1.00	
Partly working, partly retired		0.85	0.59-1.23
Unemployed or laid-off		1.55	1.30-1.84
Student		1.34	1.03-1.73
Parental leave/stay at home parent		1.06	0.77-1.45
Long-term sick leave (over 6 months)		1.38	0.94-2.04
Retired		0.59	0.45-0.77
Other or out of work for other reasons		1.50	1.21-1.88
Employment industry	6435 (100)		
Retail		1.00	
Hospitality		1.38	1.13-1.70
Property maintenance		1.31	1.02-1.68
Other (including security and hairdressing)		1.10	0.88-1.37
Missing data	3631 (56)	1.50	1.31-1.71
Earned income in state taxation (monthly)†	6429 (100)		
0-999€		2.27	1.85-2.78
1000-1599€		1.73	1.48-2.03
1600-1999€		1.38	1.18-1.62
2000-2499€		1.19	1.03-1.37
2500€+		1.00	
Current transfers received (monthly)†	6429 (100)		
0-9€		1.00	
10-199€		1.12	0.97-1.30
200-399€		1.41	1.18-1.70
400-799€		1.59	1.36-1.86
800-1200€		1.40	1.16-1.68
1200€+		1.59	1.34-1.88
How well can households cover expenses with income?	6435 (100)		
With great difficulty		14.67	10.50-20.50
With difficulty		8.02	6.02-10.69
With small difficulties		3.82	2.93-4.98
Quite easily		2.06	1.57-2.70
Easily		1.47	1.10-1.96
Very easily		1.00	

*Univariate binary logistic regression analysis
†Data from 2018, all other data from 2019.
