# Supplementary material for: Food insecurity among Finnish private service sector workers: validity, prevalence and determinants
Source: Public Health Nutr. 2022 Jan 24;25(4):829–40. doi: 10.1017/S1368980022000209 (PMC9993037; doi:10.1017/S1368980022000209)
Supplement: Supplementary file 1 [file S1368980022000209sup001.zip › S1368980022000209sup007.pdf]

Supplement 6. Comparison of sex, age distribution and employment industry of study sample and Service Union United (PAM) members in 2019. Based on statistics provided via email by PAM (A Veirto, Research Manager, personal communication, 29 November 2021).

Sex	Study sample (n=6421), %	Service Union United (PAM), %
Female	80	76
Male	20	24

Age group	Study sample (n=6421), %	Service Union United (PAM), %
20 years and under	1	7
21-30 years	17	25
31-40 years	23	21
41-50 years	23	16
51-60 years	26	16
over 60 years	11	15

Employment industry	Study sample (n=2804), %	Service Union United (PAM), %
Retail	50	49
Hospitality	20	21
Property maintenance	12	15
Security	4	3
Managers	NA*	4
Specialised industries	NA*	3
Others	13	5

*Category was not an option in the survey.
